# Immune response to the recombinant herpes zoster vaccine in people living with HIV over 50 years of age compared to non-HIV age-/gender-matched controls (SHINGR’HIV): a multicenter, international, non-randomized clinical trial study protocol

**DOI:** 10.1186/s12879-024-09192-5

**Published:** 2024-03-19

**Authors:** Maxime Hentzien, Fabrice Bonnet, Enos Bernasconi, Emmanuel Biver, Dominique L. Braun, Aline Munting, Karoline Leuzinger, Olivier Leleux, Stefano Musardo, Virginie Prendki, Patrick Schmid, Cornelia Staehelin, Marcel Stoeckle, Carla S. Walti, Linda Wittkop, Victor Appay, Arnaud M. Didierlaurent, Alexandra Calmy

**Affiliations:** 1 HIV/AIDS Unit, Division of Infectious Diseases, Geneva University Hospitals, Geneva, Switzerland; 2 University of Reims Champagne-Ardenne, Reims, France; 3 CHU de Bordeaux, Hôpital Saint-André, Service de Médecine Interne et Maladies Infectieuses, Bordeaux, France; 4 Université de Bordeaux, INSERM, Institut Bergonié, BPH, U1219, CIC-EC 1401, Bordeaux, F-33000 France; 5 Department of Infectious Diseases, Ente Ospedaliero Cantonale, Lugano, Switzerland; 6 Division of Bone Diseases, Geneva University Hospitals, Geneva, Switzerland; 7 Division Department of Infectious Diseases and Hospital Epidemiology, University Hospital Zurich, University of Zurich, Zurich, Switzerland; 8 Service of Infectious Diseases, Centre Hospitalier Universitaire Vaudoise (CHUV), Lausanne, Switzerland; 9 Clinical Virology, University Hospital Basel, Basel, Switzerland; 10 Division of Infectious Disease, Geneva University Hospital, Geneva, Switzerland; 11 Division of Infectious Diseases and Hospital Epidemiology, Kantonsspital, St Gallen, Switzerland; 12 Department of Infectious Diseases, Bern University Hospital, University of Bern, Bern, Switzerland; 13 Division of Infectious Diseases and Hospital Epidemiology, University Hospital Basel, Basel, Switzerland; 14 CHU de Bordeaux, Service d’information médicale, INSERM, Institut Bergonié, CIC-EC 1401, Bordeaux, F-33000 France; 15 Inria équipe SISTM team, Talence, France; 16 Université de Bordeaux, CNRS UMR 5164, INSERM ERL 1303, ImmunoConcEpT, Bordeaux, 33000 France; 17 Department of Pathology and Immunology, Center of Vaccinology, Faculty of Medicine, University of Geneva, Geneva, Switzerland

**Keywords:** Herpes zoster, Recombinant zoster vaccine, AS01, Shingrix®, HIV infection, Varicella-zoster virus, Aging, Prevention, Immunogenicity

## Abstract

**Background:**

The burden of herpes zoster (shingles) virus and associated complications, such as post-herpetic neuralgia, is higher in older adults and has a significant impact on quality of life. The incidence of herpes zoster and post-herpetic neuralgia is increased in people living with HIV (PLWH) compared to an age-matched general population, including PLWH on long-term antiretroviral therapy (ART) with no detectable viremia and normal CD4 counts. PLWH – even on effective ART may- exhibit sustained immune dysfunction, as well as defects in cells involved in the response to vaccines. In the context of herpes zoster, it is therefore important to assess the immune response to varicella zoster virus vaccination in older PLWH and to determine whether it significantly differs to that of HIV-uninfected healthy adults or younger PLWH. We aim at bridging these knowledge gaps by conducting a multicentric, international, non-randomised clinical study (SHINGR’HIV) with prospective data collection after vaccination with an adjuvant recombinant zoster vaccine (RZV) in two distinct populations: in PLWH on long-term ART (> 10 years) over 50 years of and age/gender matched controls.

**Methods:**

We will recruit participants from two large established HIV cohorts in Switzerland and in France in addition to age-/gender-matched HIV-uninfected controls. Participants will receive two doses of RZV two months apart. In depth-evaluation of the humoral, cellular, and innate immune responses and safety profile of the RZV will be performed to address the combined effect of aging and potential immune deficiencies due to chronic HIV infection. The primary study outcome will compare the geometric mean titer (GMT) of gE-specific total IgG measured 1 month after the second dose of RZV between different age groups of PLWH and between PLWH and age-/gender-matched HIV-uninfected controls.

**Discussion:**

The SHINGR’HIV trial will provide robust data on the immunogenicity and safety profile of RZV in older PLWH to support vaccination guidelines in this population.

**Trial registration:**

ClinicalTrials.gov NCT05575830. Registered on 12 October 2022. Eu Clinical Trial Register (EUCT number 2023-504482-23-00).

## Background

The burden of herpes zoster (HZ) virus, also known as shingles, and associated complications such as post-herpetic neuralgia is higher in older adults and has a significant impact on the quality of life in this population [1]. HZ is caused by the reactivation of the varicella zoster virus (VZV) that remains latent in the nervous system after previous infection. Each year In the USA, HZ develops in half a million people aged 60 years and over [2]. In people living with HIV (PLWH), the incidence of shingles and post-herpetic neuralgia is significantly increased compared to an age-matched general population, even in those on long-term antiretroviral therapy (ART) with no detectable viremia and normal CD4 counts [3, 4].

A recent meta-analysis of reported HZ cases, excluding patients on immunosuppressive medication, showed a greater risk of HZ among adults with HIV/AIDS compared to controls (relative risk = 3.22; 95% CI, 2.40–4.33) [5]. While the introduction of ART reduced HZ incidence, it remains a significant issue in this population [6, 7], especially for older PLWH due to the combined impact of HIV-related immunosuppression and age-related immunosenescence [8]. Indeed, It is well established that PLWH exhibit a sustained immune dysfunction despite viral suppression under ART [9–11]. Defects in the T- and B-cell memory response have been reported, as well as in T follicular helper (Tfh) cells that are essential for the formation of a high and functional antibody response [12, 13]. The follicular architecture of the lymph node, a site where T- and B-cells interact to mount an effective antibody response to vaccines, is altered in PLWH, which may explain why these patients have less Tfh cells. Although vaccines remain generally immunogenic in PLWH in term of antibody levels there is a paucity of data assessing cellular (T and B lymphocytes)responses to vaccines s in PLWH, which may be specifically reduced compared to HIV-uninfected controls, as observed after influenza vaccination [14]. Importantly, HIV infection worsens the effect of age on the responsiveness to influenza vaccine [15, 16]. Cytomegalovirus (CMV) latent infection in these patients may also contribute to an hypo-responsiveness to vaccines [17]. In the context of HZ and zoster vaccination, whereby cellular responses are particularly important, it is therefore relevant to assess whether the magnitude and/or the quality of the VZV-specific antibody and T-cell response in older PLWH are the same as in HIV-uninfected individuals or younger PLWH.

In addition to antibody and T-cell responses, there is also evidence that innate immunity, such as monocyte function, is specifically altered in PLWH and that baseline systemic inflammation is elevated in this population [11, 18]. These mechanisms may interfere with the mode of action of vaccines relying on the activation of innate immunity, such as adjuvanted or mRNA-based vaccines [19, 20]. However, data accumulated during the H1N1 2009 pandemic with the AS03- and MF59-adjuvanted vaccines showed that adjuvanted vaccines has shown an acceptable safety profile and effective despite in PLWH [21, 22]. Although most data were generated in relatively young populations [23, 24], this may indicate that adjuvants could help to overcome the immune dysregulation in this population.

There are currently two approved zoster vaccines. The live attenuated zoster vaccine (Zostavax®, Merck & Co,) and is well tolerated and modestly increases VZV-specific antibodies in PLWH on ART [25, 26]. The adjuvanted recombinant zoster vaccine (RZV) (Shingrix®, GlaxoSmithKline,) has been approved in the USA in 2017 and more recently in the European Union and Switzerland. In Switzerland, RZV is recommended for the prevention of HZ in (a) everyone aged 65 years and older, (b) in those over 50 years old living with a current or future state of immunosuppression associated with a higher risk for HZ (such as all PLWH), and (c) in those over 18 years old who are severely immunosuppressed, such as PLWH with CD4 counts ≤ 200c/µL or < 15%. RZV is a subunit vaccine containing the VZV glycoprotein E (gE) antigen and the adjuvant system AS01_B_ (a liposomal formulation containing monophosphoryl lipid A and QS-21). RZV vaccination has an acceptable safety profile [27] and leads to high and sustained efficacy against HZ (> 84%), including in people aged 70 years and above) [28–30], and is expected to be at least cost effective [31] and potentially cost-saving [32] in aged patients (≥ 50 years) [33, 34]. It is thought that both the antibody and T-cell response to the vaccine are important to prevent reactivation of VZV and HZ [35].

The immune response to RZV is characterized by robust and long-lasting gE-specific antibodies and CD4 + T-cell responses, but with no or limited induction of CD8 + cytotoxic T-cells [36, 37]. However, only very limited data are available on the immunogenicity and safety of RZV in PLWH, especially in those with lower levels of CD4 T-cells and with additional risk factors, such as age or comorbidities. In a randomized phase II study including 123 HIV patients, RZV was shown to be immunogenic and well tolerated [38]. Although RZV induced a detectable immune response after two doses with no additional benefit of a third dose, the response was not compared to age-matched healthy adults. Of note, the mean age of patients in the study was 46.6 (range, 23–74) years and therefore, no data exist for adult PLWH > 75 years of age (YOA) who may be the least responsive to the vaccine. In addition, participants had a viral RNA load of < 40 copies/mL at baseline and most had relatively high CD4 + T-cell counts with an average of 594.31 ± 273.55 cells/µl in the entire cohort, thus leaving a data gap for those with lower residual T-cells.

Vaccination, including with non-adjuvanted vaccines, can induce a transient increase in HIV viral load, although with little or no clinical significance on the course of the HIV infection [39–41]. In Yek C et al., an increase in HIV RNA was reported in some participants in both RVZ and placebo recipients, but with no statistically significant difference between groups [41]. A better characterization of the effect of RZV on HIV viral load is warranted in different HIV populations, i.e., among the elderly [42] and those on long-term ART.

In this multicenter, international, non-randomized clinical trial we will perform an in-depth evaluation of the immunogenicity and safety profile of RZV in PLWH > 50 YOA on long-term ART (> 10 years) and compare the results across age-groups and to non-HIV age-/gender-matched controls to investigate the combined effect of aging and immunosuppression due to chronic HIV disease. The specific objectives of the study include the following: (1) To evaluate whether immune dysregulation to a long history of HIV disease/ART impacts the immunogenicity (antibody and T-cell response) of the RZV vaccine in aged PLWH compared to age-/gender-matched healthy controls; (2) To assess whether age impacts immunogenicity in PLWH; (3) To assess the benefit/risk profile of RZV vaccine in PLWH, irrespective of age, by monitoring reactogenicity and unsolicited adverse events after vaccination; (4) To determine the effect of vaccination on HIV viral load; (5) To compare inflammatory responses induced by RZV in PLHW and controls; (6) To identify pre-vaccination markers (e.g., pre-existing immunity to the vaccine antigen, CMV status, ageing immune markers) predictive of the vaccine response.

## Methods

The trial will be sponsored by the HIV Unit, Department of Infectious Diseases, Geneva University Hospitals (Geneva, Switzerland). The study is registered on ClinicalTrials.gov (NCT05575830) and on the Eu Clinical Trial Register (EUCT number 2023-504482-23-00).

### Study design and sites

**Fig. 1:**
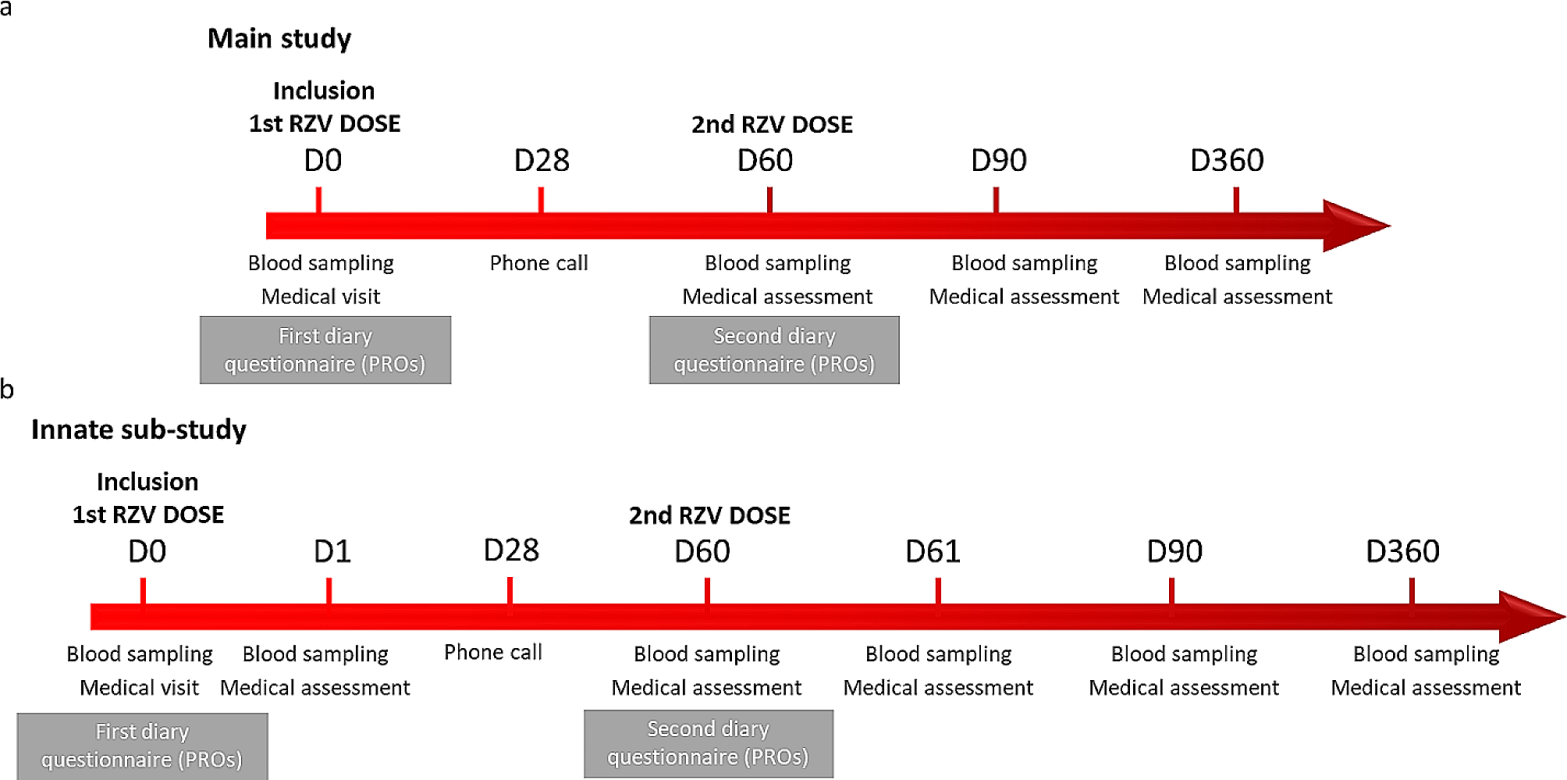
Timeline for study procedures and visits for the main study (**a**) and innate sub-study (**b**). Abbreviations: RZV, recombinant zoster virus; D, day

The SHINGR’HIV trial is a multicentric, international non-randomized clinical study with prospective data collection after vaccination with an adjuvant recombinant zoster vaccine (RZV) in two distinct populations: PLWH on long-term ART (> 10 years) over 50 years of age and age/gender matched controls (phase IV - post marketing in Switzerland and phase II studies in PLWH and non-HIV infected controls in France because at the time of protocol approval, the vaccine was not commercially available in France). Participants will be recruited at seven clinical sites in Switzerland and at one site in France. The clinical study will have two groups, PLWH and non- HIV infected control participants, who will receive two doses of RZV two months apart. Participants will be enrolled in the main study, which includes four visits with four blood draws (Fig. 1a). Consenting participants will have the possibility to be included in the innate immunity sub-study, which includes two additional visits and blood sampling on the day after each vaccine dose (Fig. 1b). The trial will be conducted at the following institutions: Geneva University Hospitals; University Hospital Basel; Bern University Hospital; University Hospital of Lausanne; Lugano Cantonal Hospital; Kantonsspital St. Gallen, and Zurich University Hospital in Switzerland; and Bordeaux University Hospital in France.

### Study population and recrutement

**Fig. 2:**
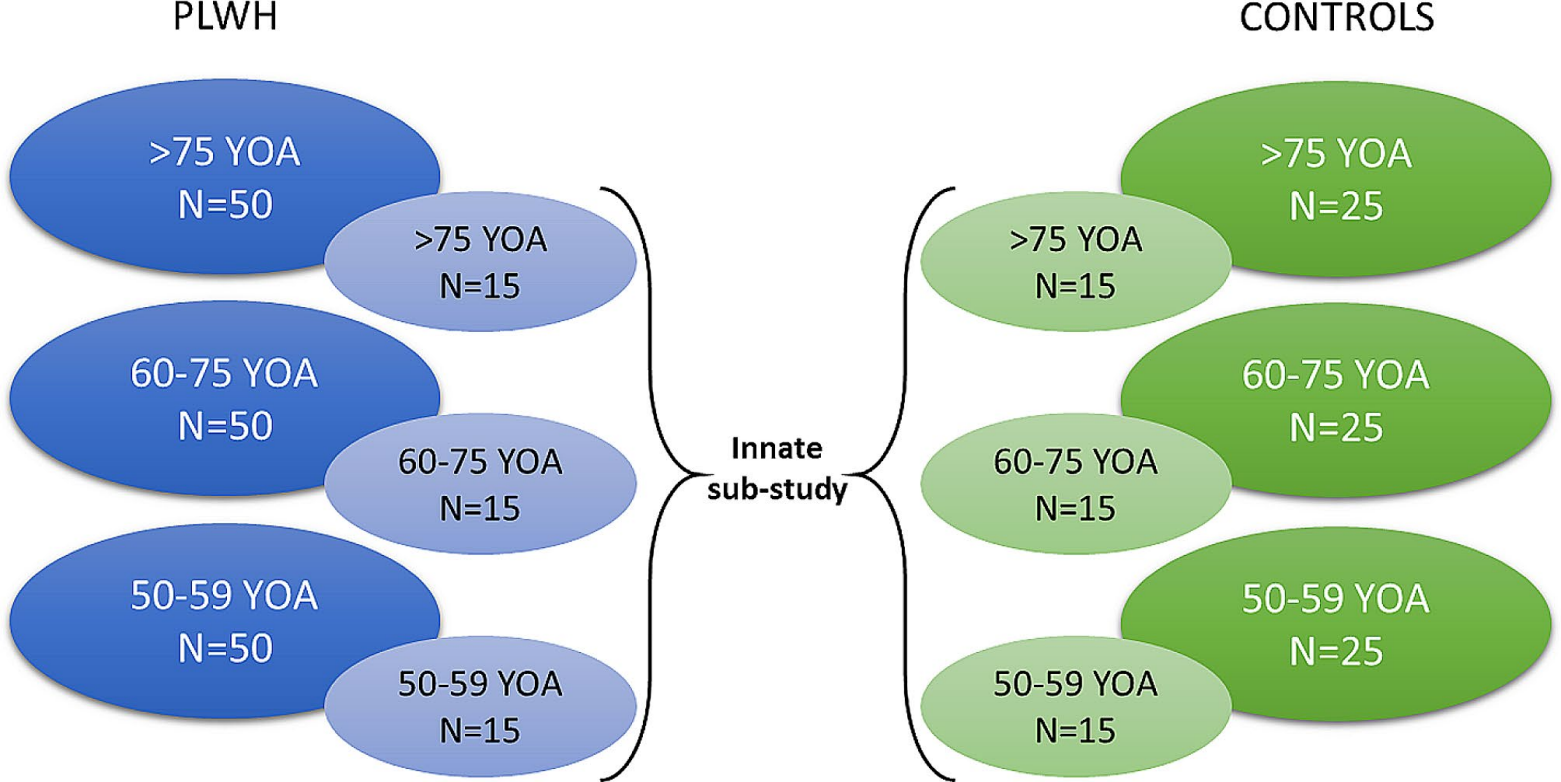
Stratification of the study population according to the age (YOA). *Abbreviations* PLWH, people living with HIV; YOA, years of age

The study will consist of six groups (Fig. 2). PLWH participants will be stratified in three age groups (*N* = 50 per group): >75 YOA; between 60 and 75 YOA; and between 50 and 59 YOA. Gender-matched healthy adults will be stratified following the same age ranges (*N* = 25 per group). Gender matching will be performed as follows: for every two men or women included in one age strata in the PLWH group, one man or one woman in the same age strata will be included in the control group. At least fifteen participants of each group will be included in the innate sub-study PLWH will be recruited from two large established HIV cohorts: the Swiss HIV cohort study (SHCS) and the French National Agency for AIDS Research (Agence Nationale de la Recherche sur le SIDA et les hépatites virales [ANRS]) CO3 Aquivih-NA cohort in Aquitaine. This gives access to bio-banked biological specimens and the medical history of the participants of the study. Controls participants will be recruited from distinct sources: (1) Patients followed at the Bordeaux University Hospital’s multidisciplinary health center, a multi-professional medical center attached to the CHU de Bordeaux; (2) Healthy volunteer cohort of the Bordeaux University Hospital; (3) Participants already followed as part of the GERICO cohort, which is the Geneva Retiree Cohort, recruited from the general population and prospectively followed to assess their bone health evolution.

### Eligibility criteria

Inclusion criteria for PLWH participants are: (1) already registered in the SHCS or ANRS CO3 Aquitaine cohort; (2) age > 50 YOA; (3) time since first ART initiation > 10 years; d) not already vaccinated with RZV; (4) HIV viral load < 50 copies/ml; (5) informed consent as documented by signature; and (only for French candidates) person affiliated with or beneficiary of the French social security scheme. Inclusion criteria for age-/gender-matched controls are: (1) age > 50 YOA; (2) not already vaccinated with RZV; (3) informed consent as documented by signature; (4) not HIV infected; and (5) (only for French candidates) person affiliated with or beneficiary of the French social security scheme.

Participants will be excluded if they meet any of the following criteria: (a) ongoing signs of febrile or non-febrile infection at the time of the first vaccination; (b) Immunosuppression due to current malignant neoplasm, primary immunodeficiency or recent (< 2 years) solid or bone-marrow transplant or any transplant still requiring immunosuppressive therapy; (c) intake of drugs that suppress the immune system (e.g., glucocorticoids over a long period of time [an equivalent dose of prednisone > 20 mg/day > 3 months]), monoclonal antibodies, cytostatics, biological products) within 6 months prior to enrollment; (d) having received any other vaccine than RZV in the last month or expected to receive any vaccine other than RZV in the following month; (e) having received a shingles vaccine within one year; (f) presented with HZ in the previous year; (g) contraindication to RZV; (h) hospitalized patients; (i) unable to provide informed consent or inability to follow the study procedures, e.g. due to language problems, psychological disorders, dementia, etc.; (j) participation in another study with an investigational drug within the 30 days preceding study entry or during the study; k) pregnant or breastfeeding woman; and l) (only for French candidates) patient governed by articles L 1121-5 to L 1121-8 (persons deprived of their liberty by a judicial or administrative decision, minors, persons of legal age who are the object of a legal protection measure or unable to express their consent).

### Intervention

Consenting participants will receive two doses of RZV two months apart. Each dose contains 50 µg of VZV glycoprotein E (gE), produced in Chinese hamster ovary cells by recombinant DNA technology, the adjuvant AS01_B_ containing 50 µg of a purified plant extract *Quillaja saponaria Molina*, fraction 21 (QS-21), and 50 µg of the Toll-like receptor 4 ligand 3-O-desacyl-4’-monophosphoryl lipid A (MPL) from *Salmonella Minnesota* [35]. RZV will be prepared and injected according to the manufacturer’s instructions. No dosage modification is expected for study participants. The second dose of the vaccine may not be given in the case of a contraindication appearing after the first dose (e.g. hypersensitivity reaction). The patient may decide to withdraw his/her participation at any time.

### Outcome measures

The primary outcome is the geometric mean titer (GMT) of gE-specific total IgG measured at day (D) 90 (1 month after the second vaccine dose) by ELISA. Secondary outcomes will include: mean of gE-specific CD4 + T-cells expressing at least two activation markers (i.e., a combination of the following markers: CD40L, intracellular IFN-gamma, IL-2 or TNF-alpha) after activation by a pool of peptides covering the entire gE (ref: 31) and measured at D90; kinetics of the GMT of gE-specific IgG measured at D0, D60, D90 and D360; kinetics of the gE-specific CD4 + T-cells expressing at least two activation markers measured at D0, D90, and D360; vaccine response rate for antibody defined as the percentage of individuals with a ≥ 4-fold increase in the anti-gE antibody concentration compared to the pre-vaccination concentration (for initially seropositive participants) or compared to the anti-gE antibody cut-off value for seropositivity (for initially seronegative participants); and the vaccine response rate for T-cells defined as the percentage of individuals with a ≥ 2-fold increase in the frequency of specific CD4 + T-cells compared to pre-vaccination frequencies or a ≥ 2-fold increase above the cut-off (for participants with pre-vaccination frequencies below the cut-off).

Changes in the level of serum pro-inflammatory markers (including a panel of cytokines and c-reactive protein) between D0 and D1 (first dose) and between D60 and D61 (second dose) will be measured using a multiplex assay for the innate sub-study. The differential expression of innate and adaptive immune response genes and associated pathways at D1 and D60 compared to D0 (first dose) and at D61 and D90 compared to D60 (second dose) will be assessed by RNAseq. Samples will be used for a more in-depth exploratory analysis of the immune response and the identification of potential clinical or biological markers associated with the vaccine response. For example, we will measure changes in the activation and memory markers on gE-specific T- and B-cells by flow cytometry and in the functionality of antibodies using specifically designed assays. We will also investigate biomarkers at baseline such as CMV serological status, and CD4 T-cell count,associated with a potential reduction in vaccine response.

Safety outcomes will include the incidence of solicited local and systemic adverse events (AEs) in the 7 days following each vaccine dose (reactogenicity) collected in a diary questionnaire (Table 1). Unsolicited AEs will be collected for 28 days after each vaccine dose. Serious AEs and the incidence of potential immune-mediated disorders [43] will be collected throughout the study period. Finally, given the historical evidence of the effect of vaccines on HIV viral load, the percentage of PLWH with a HIV viral load > 50 copies/ml will be measured 1 month after the second vaccine dose (D90).

**Table 1:** Adverse events diary questionnaire

**Local symptoms**	**Pain at injection site**
	Injection site swelling
	Injection site redness
	Injection site itching
	Axillary swelling and tenderness of the vaccination arm
	Other local symptoms
**Systemic symptoms**	Fatigue
	Headache
	Muscle ache
	Joint pain
	Chills
	Nausea/vomiting
	Diarrhea
	Fever
	Dizziness
	Other symptoms

The questionnaire can be completed either as a hard copy or electronically through RedCap and will be distributed at D0 and D60

### Visit schedule

Potential eligible participants will be screened by a phone call or during a routine hospital visit at least 7 days before study inclusion to allow sufficient time to provide consent to study participation. At the inclusion visit (D0), written consent will be collected before starting any procedure. For the main study, there will be four on-site visits (D0, D60, D90, D360) and one phone call (D28) (Fig. 1a), while for the innate sub-study there will be six on-site visits (D0, D1, D60, D61, D90, D360) and one phone call (D28) (Fig. 1b). Vaccine doses will be given at D0 and D60 ± 7 days. If a temporary contra-indication occurs, the vaccine could be delayed at the shortest interval possible, but up to 6 months after the first injection.

### Justification of sample size

We hypothesize that anti-gE specific IgG GMT at D90 will differ between the PLWH group and the non-HIV group and assume that a GMT difference of 25–30% is clinically relevant. Based on data from 1070 healthy individuals of 50 YOA and older available in the ‘Summary of Product Characteristic’(44), we assumed a reference value of anti-gE specific IgG geometric mean concentration in the control group of 52377 mUI/mL with an upper limit of the 95% CI of 54578 mUI/mL. The standard deviation of the natural log of the mean was 0.68. Given the fact that we can expect a more heterogeneous response in PLWH, we increased the standard deviation by 20% to 0.82 natural log mUI/mL. Using a two-tailed parametric test (t-test; SAS Proc POWER two sample means) with β = 0.2 (80% power), α = 0.05, a sample size of 150 in the PLWH group and 75 in the non-HIV group will allow to detect a difference of 14649.5 mUI/mL for anti-gE antibodies (representing 28% of the mean value measured in healthy adults). Based on data from the above-mentioned study, this sample size will also allow to have 80% power to detect a difference in T-cell responses of 600 cells per million T-cells (representing 32% of the mean value observed in healthy adults). Assuming 5% loss to follow-up at D90, we would be able to demonstrate a difference of 14995 mUI/mL for anti-gE antibodies and a difference of 616 cells per million T cells between PLWH and non-HIV infected controls.

### Statistical analysis

Geometric means and their 95% CIs of gE-specific IgG (primary endpoint) and secondary endpoints will be calculated and graphically described in the PLWH and non-HIV groups and according to age groups. Several statistical approaches (i.e., linear and logistic regression, mixed models, survival models and integrative methods) will also be considered for the analysis of the primary and secondary endpoints. All statistical tests will be two-sided with a significance level of 0.05. Two-sided 95% CIs will be reported. Linear regression models will be adjusted on potential confounders and determined by directed acyclic graphs using the available literature. Baseline anti-gE level, age, gender, ethnicity, and history of shingles will also be considered in the adjusted model. As a secondary analysis, we will add an interaction term, if possible, in the model, to assess for an interaction between age and HIV status. Finally, an integrative approach of all available data generated in this study will also be considered using statistical approaches to relate and down-select several markers among the high-dimensional data, i.e., sPLS or similar techniques. As a sensitivity analysis, we will use multiple imputation to account for missing data. Very few missing data are expected due to the study design, the population studied, and the fact that patients are already participating in cohort studies.

### Data collection, management and monitoring

Data will be collected on hard copy case report forms (CRF) by the clinical investigator or designated site staff and entered in the database REDCap™, a certified Good Clinical Practice-compliant electronic clinical data management system used to develop an electronic CRF (eCRF). The eCRF will reflect the study plan and all subject-related datato be collected, including primary immunological and safety outcomes. Data and metadata will be exported from REDCap™ in plain text (CSV, SPSS) for analysis and the entire database will be archived for a minimum period of 15 years. The REDCap™ platform ensures traceability and safety. Data will be stored on identified servers allowing for safety back-ups as needed. Biological material will be identified by a unique participant number and only accessible to authorized personnel. Participant data collected throughout the duration of the study can be analyzed with data from other research groups. For quality control of the study conduct and data collection, all study sites will be monitored by appropriately trained and qualified personnel according to ICH Good Clinical Practice guidelines.

## Discussion

In this prospective, multicentre, international open-label phase IV (Switzerland) and phase II (France) clinical trial, we will perform an in depth-evaluation of the humoral, cellular and innate immunogenicity and safety profile of RZV in PLWH over 50 YOA on long-term ART (> 10 years) compared to non-HIV age-/gender-matched controls to address the combined effect of aging and potential immune deficiencies due to chronic HIV infection. Importantly, the profile of the vaccine response in older PLWH will be, for the first time to our knowledge, compared to healthy age-/gender-matched controls. Patients will be recruited from two large HIV cohorts, established for several years in Switzerland and in France, with access to their bio-banked biological specimens and medical history. These data are expected to set the foundation for future studies. We expect to be able to monitor vaccine effectiveness in these two patient cohorts and potentially define immune responses associated with vaccine breakthrough by taking advantage of the bio-banked samples routinely collected in the cohort. Addressing whether RZV remains highly immunogenic with an acceptable safety profile in older PLWH is critical and will support the development of vaccination guidelines in this population. This study is therefore expected to lead to significant contributions to the field and ultimately improve prevention of HZ in PLWH and their quality of life and provide important data for general vaccine response in PLWH.

### Trial status

The trial is funded both in Switzerland and in France. The recruitment process has been active in Switzerland since December 2022, and in France since November 2023. Suisse protocol version 2.0 of 21/09/2022, France protocol version 1.1 of 26/06/2023.

## Data Availability

No datasets were generated or analysed during the current study.

## Abbreviations

AE: adverse events
ART: antiretroviral therapy
CMV: cytomegalovirus
CRF: case report form
GMT: geometric mean titer
HZ: herpes zoster
PLWH: people living with HIV
RZV: recombinant zoster vaccine
SHCS: Swiss HIV Cohort Study
Tfh: T follicular helper (cells)
VZV: varicella-zoster virus
YOA: years of age
gE: glycoprotein E
